# Relationships among types of activity engagement and insomnia symptoms among older adults

**DOI:** 10.1186/s12877-021-02042-y

**Published:** 2021-01-30

**Authors:** Da Eun Kim, Tonya J. Roberts, Chooza Moon

**Affiliations:** 1 College of Nursing and Research Institute of Nursing Science, Kyungpook National University, Daegu, Republic of Korea; 2 School of Nursing, University of Wisconsin-Madison, Madison, WI USA; 3 College of Nursing, University of Iowa, Iowa City, IA USA

**Keywords:** Sleep, Leisure activities, Exercise, Aged

## Abstract

**Background:**

An increasing awareness exists that lack of activity engagement is associated with insomnia symptoms. However, the majority of studies have focused on the association between a single type of activity engagement and insomnia symptoms.

**Methods:**

This is a cross-sectional study using secondary data from the Health and Retirement Study examining the relationships among different types of activity engagement and insomnia symptoms among older adults. The sample for this study included 3321 older adults who responded to survey modules on activity engagement and insomnia symptoms in 2016. Activity engagement was measured using items for three types of activities (i.e., social, cognitive, and physical) validated in this study. Insomnia symptoms were measured using four items (i.e., difficulty of falling asleep, waking up during the night, waking up too early, and feeling rested). Independent *t*-tests were conducted to identify the differences in insomnia symptoms according to activity engagement level. Regressions were conducted to examine the associations among three types of activity engagement and insomnia symptoms after adjusting for covariates such as demographics, chronic disease, activities of daily living difficulty, cognitive function, sleep disorder, loneliness, and caregiving.

**Results:**

The respondents in the high-level social, cognitive, and physical activity engagement groups were found to show fewer insomnia symptoms. Furthermore, higher social (*β* = − 0.04, *p* = 0.040) and cognitive (*β* = − 0.06, *p* = 0.007) activity engagements were associated with fewer insomnia symptoms even after adjusting for other types of activity engagement and all covariates.

**Conclusions:**

This study suggests that older adults with higher social and cognitive activity engagements may be likely to have fewer insomnia symptoms. Based on these results, future research is needed to develop multi-component intervention programs that can encourage older adults to engage in these activities.

**Supplementary Information:**

The online version contains supplementary material available at 10.1186/s12877-021-02042-y.

## Background

Insomnia symptoms are recognized as a significant symptom in older adults. Insomnia symptoms are defined as complaints of disturbed sleep including difficulty initiating sleep, difficulty maintaining sleep, early morning awakening, and nonrestorative or poor-quality sleep [1]. Insomnia symptoms are more prevalent in older adults than in other age groups, and approximately 50% of older adults report these symptoms [2]. Insomnia symptoms are associated with adverse outcomes such as frailty and poor physical and mental health quality of life [3, 4]. Furthermore, people with insomnia symptoms are more prone to cardiovascular disease, neurologic disease, and pain [5]. Therefore, fully understanding the factors that influence insomnia symptoms among older adults is imperative.

Evidence increasingly shows that insomnia symptoms in older adults are associated with a lack of activity engagement [6]. According to the two-process model of sleep regulation, activity is related to two internal biological sleep–wake mechanisms. The first one is the circadian rhythm which synchronizes the sleep–wake rhythm with local time based on external stimuli [7]. Structured schedules of activity during the daytime serve as external time cues (i.e., Zeitgebers) to continuously regulate the sleep–wake rhythm [7] and increase slow-wave sleep [8]. The second one is sleep–wake homeostasis, which is maintained by the generation of homeostatic sleep pressure or sleep need accumulated by wakefulness during the daytime [7]. In particular, physical activity may increase sleep-promoting substance adenosine, which increases the homeostatic drive to sleep [9]. Furthermore, activity engagement may be a modifiable factor that could be targeted for developing interventions to reduce insomnia among older adults. However, the relationship between activity engagement and insomnia symptoms must be better understood.

The Senescent Sleep Model, a conceptual model for describing factors influencing sleep complaints in older persons, states that multiple factors including physical and psychosocial factors affect sleep complaints in different ways [10]. Activity engagement is one factor that can be related to sleep. Engaging in different types of activities can affect sleep through different mechanisms. For example, social activity may reduce loneliness [11] and stress [12], which can affect sleep complaints [13]. Moreover, cognitive activity can provide cognitive stimulation that may reduce sleep complaints among older adults [14]. Also, physical activity can lead to an increase in core body temperature, which can facilitate initiation of sleep [15] and improve sleep through the anxiolytic effects of physical activity [16]. Furthermore, physical activity, which may lead to energy expenditure, requires body restoration and better sleep [17].

Accordingly, there is a growing body of research examining the association of sleep and different types of activity engagement. A previous study has concluded that social participation including religious services, volunteer work, and organized group meetings is positively associated with sleep patterns among older adults [18]. Cognitive activity such as playing games and knitting or sewing shows a significant association with fewer sleep disturbances [19]. In addition, more physical activity engagement is associated with higher sleep quality and better sleep patterns in older adults [20, 21]. However, the majority of previous studies have focused on a single type of activity when they examined the relationship between activity engagement and sleep complaints among older adults. The association of insomnia symptoms with engagement in multiple types of activities should be examined together because older adults engage in various types of activities in their daily lives. It can contribute to a more holistic understanding of the impact of activity engagement on insomnia symptoms.

The overall purpose of this study is to examine the relationships among different types of activity engagement and insomnia symptoms in older adults. The specific aims are to compare insomnia symptoms according to the types of activity engagement (i.e., social, cognitive, and physical) and to identify the relationships among different types of activity engagement and insomnia symptoms after controlling for other types of activity engagement in community-dwelling older adults.

## Methods

### Study design and dataset

This study is a cross-sectional secondary data analysis using a dataset derived from a population-based longitudinal study, the Health and Retirement Study (HRS). The HRS is sponsored by the National Institute on Aging (grant number NIA U01AG009740) and conducted by the University of Michigan, Ann Arbor, MI, USA. Data can be obtained from the HRS website, https://hrs.isr.umich.edu. HRS data are collected every 2 years beginning in 1992 from Americans who are not institutionalized and are over the age of 50 years. Further information about the study design has been published by Sonnega et al. [22]. The core dataset collected in 2016 was used to conduct data analysis in the current study.

### Sample

The inclusion criteria for the present study were participants who are ≥65 years of age and who responded to the items for activity engagement in psychosocial and lifestyle questionnaires and items for insomnia symptoms in 2016. The core HRS interviews, which include items about insomnia symptoms, have been conducted every 2 years since 1992. In the case of items for activity engagement, the HRS started to collect activity data as part of the psychosocial and lifestyle questionnaires in the core dataset from a randomly selected sample of 50% of the participants in 2006. Those who were interviewed in 2006 responded to the questionnaires again in 2010 and 2014 [23]. The remaining 50% of the participants have been administered psychosocial and lifestyle questionnaires every 4 years beginning 2008 (i.e., 2008, 2012, and 2016). Thus, the participants who were interviewed for the activity engagement items in 2016 were about 50% of the total participants in the core HRS interviews. The final sample in this study included 3321 persons.

### Measurements

#### Insomnia symptoms

Respondents were asked about their insomnia symptoms using four items: 1) how often trouble falling asleep, 2) how often trouble with waking up during the night, 3) how often trouble with waking up too early and not being able to fall asleep again, 4) how often feel really rested when waking up in the morning (nonrestorative sleep). Each item was scored on a three-point Likert scale from 1 (most of the time) to 3 (rarely or never). The first three items were reverse-coded and a summed total score was then calculated based on the coding methods in a previous study [24]. The total insomnia symptom score ranged from 3 to 12, with a higher score indicating more insomnia symptoms.

#### Activity engagement

In the HRS, respondents were asked how often they engaged in different types of activities using 21 items in the HRS psychosocial and lifestyle questionnaires. Each item was scored on a Likert scale of 1 (never) to 7 (daily). The data showed skewness (range, − 2.69–4.97) and non-normality (*p* <  0.001) when the Shapiro–Wilk test was conducted, which indicated that the midpoint response categories (e.g., 3, 4, and 5) may not provide sufficient information. The response categories were collapsed to 1 (never), 2 (not in the last month–several times a week), and 3 (daily) because *never* or *daily* engaging in activities indicates either a clear absence of engagement or frequent engagement [25]. The exploratory factor analysis (EFA) was then performed with the oblimin rotation to examine the factor structure of the 21 items. The weighted least square mean and variance (WLSMV) adjusted estimation was used considering that the variables are categorized. In the EFA, the number of factors with eigenvalues greater than 1.00 was six (6.29, 1.71, 1.44, 1.33, 1.22, and 1.03, respectively). In the cases of four, five, and six-factor models, at least one factor did not have more than three items. Therefore, a three-factor model was determined as the final factor structure of the activity engagement questionnaires (see Additional file 1): social activity (six items: volunteer work with children, charity work, educational course, sport or social club, nonreligious organization, and community arts group), cognitive activity (five items: word games, cards or chess, writing, knitting, and hobby or project), and physical activity (three items: maintenance or gardening, playing sports or exercise, and walking for 20 min). In the three-factor model, seven items which did not adequately load (< 0.40) were excluded. The construct validity of the three-factor structure with a total of 14 activity items was verified using confirmative factor analysis. Results indicated that this three-factor model was appropriate with an acceptable fit (comparative fit index = 0.968, Tucker–Lewis index = 0.960, RMSEA = 0.066, and SRMR = 0.048) [26]. The more sensible index of internal consistency, coefficient omega, which adheres to the congeneric model to estimate reliability, was calculated because the assumptions of the essentially tau-equivalent model were not met in the data of the current study [27]. Coefficient omega was 0.89, 0.72, and 0.68 for social, cognitive, and physical activities, respectively. This indicates an acceptable reliability (≥ 0.60). The mean score of the items in each subscale, ranging from 1 to 3, was calculated. A higher score indicated higher activity engagement.

#### Covariates

Variables that are recognized as precipitating and perpetuating risk factors for insomnia symptoms were included as covariates in this study based on the Senescent Sleep Model [10], a model that has been used in many studies to identify the key factors that influence sleep among older adults. Precipitating factors included were 1) chronic diseases including heart disease, stroke, Alzheimer’s disease, dementia, hypertension, pain, arthritis, cancer, diabetes, and incontinence [24]; 2) difficulty with activities of daily living (ADL); 3) cognitive function; and 4) primary sleep disorders (e.g., sleep apnea, restless legs, and other diseases). Respondents were classified as having ADL difficulty if they reported difficulties in performing one or more of the following basic tasks: dressing, walking across a room, bathing, eating, getting in or out of bed, and toileting as defined by Katz and Akpom [28]. Cognitive function was measured by a modified version of the Telephone Instrument for Cognitive Status (TICS). This instrument consists of (a) an immediate word recall test (10 points), (b) a delayed recall test (10 points), (c) a serial 7 s subtraction test (5 points), (d) a backward counting test (2 points), (e) naming tasks (e.g., date and naming the president and vice president; 6 points), and (f) vocabulary questions (2 points). The total cognitive function score was calculated by summing all points, which ranges from 0 to 35. A lower total score indicates lower cognitive function. A total score of 8 or less was suggested as the cutoff score for cognitive impairment [29].

Loneliness and caregiving were the perpetuating factors included. Loneliness was measured using 11 items drawn from the revised UCLA Loneliness Scale [30]. Each item was rated on a Likert scale of 1 (hardly ever or never) to 3 (often). Each score, ranging from 1 to 3, was averaged with a higher score indicating greater loneliness. Caregiving was measured using one dichotomous question (0, never; 1, at least sometimes), “How often do you care for a sick or disabled adult?” Demographic variables such as age, sex, race, marital status, and education level were included.

### Statistical analysis

Independent *t*-tests were used to analyze the differences in insomnia symptoms according to the general characteristics and each type of activity engagement. In case of activity engagement, the respondents were classified into two groups according to the median value of each type of activity engagement. For example, respondents with higher and lower social activity engagements than the median value of social activity engagement scores (≥ 1.33 and < 1.33 points, respectively) were classified into the high and low social activity engagement groups, respectively. In addition, the median values of the cognitive and physical activity engagement scores were 1.60 and 2.00 points, respectively.

Multiple linear regressions were conducted to examine the associations among the different types of activity engagement and insomnia symptoms, controlling for covariates. The covariates were included step-by-step according to the type of covariates (e.g., demographics, precipitating factors, and perpetuating factors) to examine the changes in the relationship between activity engagement and insomnia symptoms caused by adjusting for covariates. The maximum likelihood estimation with the robust standard error (MLR) was used to adjust the non-normal distribution of the dependent variable and deal with missing values. Ordinal logistic regressions were also conducted to examine the association of the different types of activity engagement with each insomnia symptom, considering that each insomnia symptom is an ordered categorical-dependent variable (i.e., most of the time, sometimes, and rarely or never).

Descriptive statistics and independent *t*-test were performed using IBM SPSS Statistics, ver. 26.0 (IBM Corp., Armonk, NY, USA). Factor analyses, calculation of omega coefficient, multiple linear regressions, and ordinal logistic regressions were performed using Mplus ver. 8.3 (Muthén & Muthén, Los Angeles, CA, USA).

## Results

### General characteristics of the sample

Table 1 describes the general characteristics of the sample. The mean age of the 3321 older adults was 75.62 years (range, 65–98), and almost 60% were female. Most respondents were Caucasian (80.7%) and almost 55% were married. Also, approximately 48.9% graduated from college or had higher educational attainment. The average number of chronic diseases was 3.11 (range, 0–9). Almost 81% of the respondents reported no difficulty with any ADL tasks. The average score of the modified version of the TICS for the cognitive function was 21.54 (range, 1–34). Moreover, approximately 13.1% had sleep disorders (e.g., sleep apnea, restless legs, or other). The average score for loneliness was 1.52 (range, 1–3). Almost one-third of the respondents (30.9%) were caregivers for adults.

**Table 1:** Descriptive characteristics of the sample (*N* = 3321)

Variables	Categories	***n*** (%)	Mean (SD)	Range	Insomnia symptom score	
					Mean (SD)	***t*** (***p***)
Age (years)			75.62 (7.15)	65–98		
	65 to 74	1504 (45.3)			6.71 (2.10)	−0.23 (0.819)
	75 or older	1817 (54.7)			6.73 (2.02)	
Sex	Male	1317 (39.7)			6.46 (2.04)	6.01 (< 0.001)
	Female	2004 (60.3)			6.89 (2.05)	
Race	Caucasian	2679 (80.7)			6.67 (2.04)	2.72 (0.007)
	Other	639 (19.3)			6.92 (2.11)	
Marital status	Married	1829 (55.1)			6.55 (2.02)	5.34 (< 0.001)
	Other (e.g., separated, divorced, widowed, or never married)	1489 (44.9)			6.93 (2.08)	
Level of education	High school or lower	1695 (51.1)			6.92 (2.05)	5.71 (< 0.001)
	College or higher	1619 (48.9)			6.51 (2.05)	
Number of chronic diseases			3.11 (1.61)	0–9		
	None	141 (4.2)			5.93 (1.61)	−5.89 (< 0.001)
	1 or more	3179 (95.8)			6.76 (2.07)	
ADL difficulty	One or more difficulties	626 (18.9)			7.49 (2.16)	−9.97 (< 0.001)
	No difficulty	2689 (81.1)			6.55 (1.99)	
Cognitive function			21.54 (4.90)	1–34		
	Cognitive impairment (modified TICS score ≤ 8)	35 (1.1)			6.54 (1.82)	0.52 (0.604)
	No cognitive impairment (modified TICS score > 8)	3265 (98.9)			6.72 (2.06)	
Sleep disorder	Sleep disorder	431 (13.1)			6.94 (2.23)	−2.22 (0.027)
	No sleep disorder	2860 (86.9)			6.68 (2.03)	
Loneliness			1.52 (0.42)	1–3		
	High level (> 1.45)^a^	1489 (45.8)			7.16 (2.11)	−11.51 (< 0.001)
	Low level (≤ 1.45)^a^	1761 (54.2)			6.34 (1.93)	
Caregiving	At least sometimes	1002 (30.9)			6.70 (2.00)	0.30 (0.767)
	No	2241 (69.1)			6.73 (2.09)	
Activity engagement	Social activity		1.39 (0.34)	1–3		
	Cognitive activity		1.63 (0.38)	1–3		
	Physical activity		1.95 (0.45)	1–3		
Insomnia symptom score			6.72 (2.06)	4–12		

*ADL* activities of daily living, *SD* standard deviation, *TICS* telephone instrument for cognitive status

There are several variables with missing values including race, marital status, number of chronic diseases, ADL difficulty, cognitive function, sleep disorder, loneliness, and caregiving.

^a^Total respondents were classified into two groups (high level vs. low level) according to the median value of the revised UCLA loneliness score (1.45)

### Activity engagement and insomnia symptoms of the sample

**Fig. 1:**
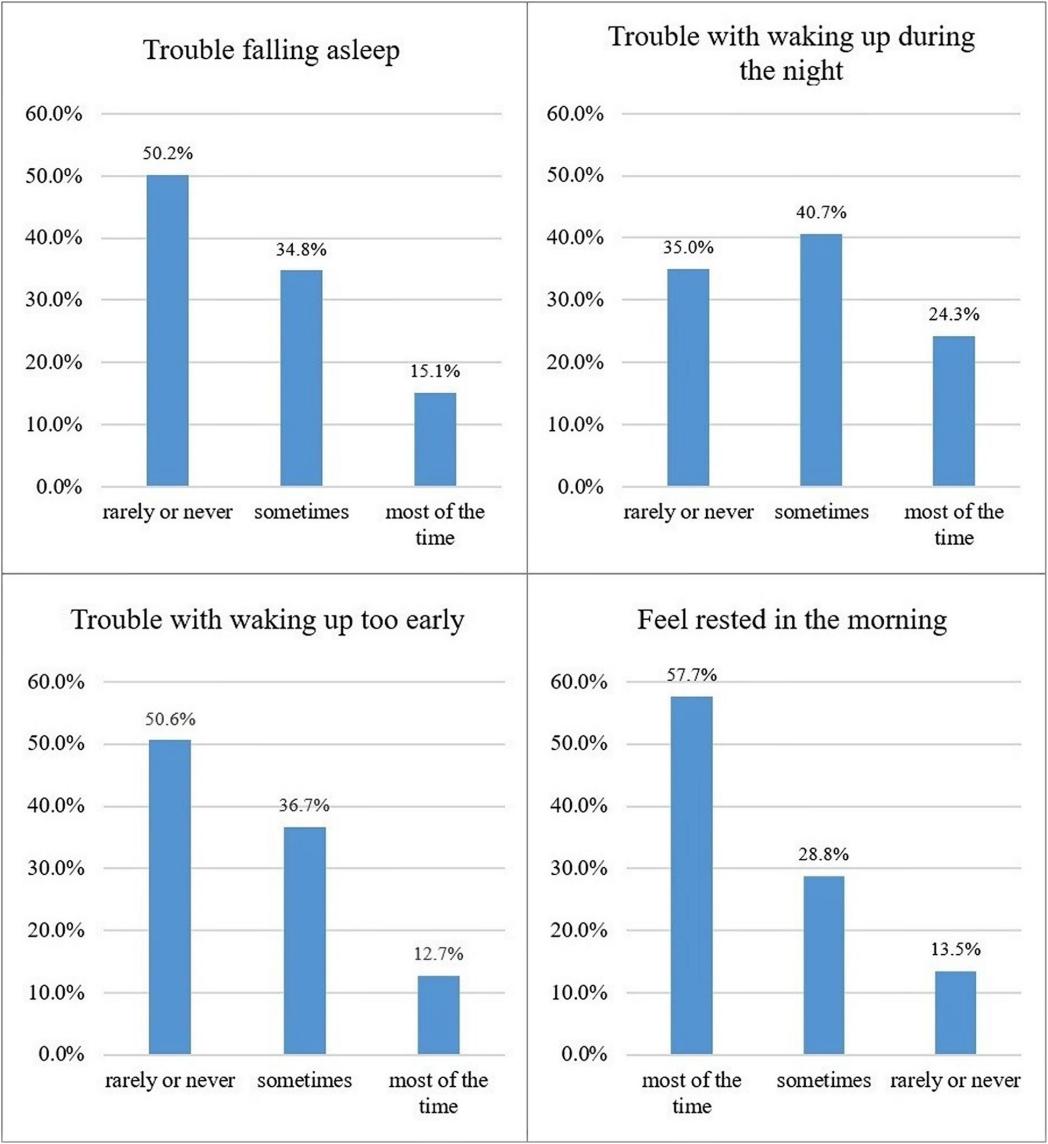
Histograms of each insomnia symptom

The overall activity engagement and insomnia symptoms of the sample were examined (Table 1). The mean activity engagement scores for the social, cognitive, and physical activity items were 1.39 (range, 1–3), 1.63 (range, 1–3), and 1.95 (range, 1–3), respectively. The mean score for insomnia symptoms was 6.72 (range, 4–12). Histograms of each insomnia symptom were presented in Fig. 1.

### Comparison of insomnia symptoms according to general characteristics

The insomnia symptoms were compared according to the general characteristics of the respondents (Table 1). Respondents who are male, Caucasian, and married showed fewer insomnia symptoms. However, respondents who graduated from high school or lower and had at least one chronic disease reported more insomnia symptoms. Furthermore, respondents with one or more ADL difficulty and sleep disorder and those in the high-level loneliness group showed more insomnia symptoms.

### Comparison of insomnia symptoms by each type of activity engagement

The comparison of the insomnia symptoms by levels of each type of activity engagement is presented in Table 2. The independent *t*-test showed that the respondents with higher social activity engagement (*t* = 7.43, *p* <  0.001), higher cognitive activity engagement (*t* = 5.57, *p* <  0.001), and higher physical activity engagement (*t* = 7.20, *p* <  0.001) had fewer insomnia symptoms.

**Table 2:** Comparison of insomnia symptoms by each type of activity engagement (*N* = 3321)

Types of activity	Group	Insomnia symptoms		
		Mean (SD)	***t***	***p***
Social activity	High-level group (*n* = 1833)	6.48 (2.00)	7.43	< 0.001
	Low-level group (*n* = 1479)	7.01 (2.09)		
Cognitive activity	High-level group (*n* = 2008)	6.56 (2.02)	5.57	< 0.001
	Low-level group (*n* = 1306)	6.96 (2.10)		
Physical activity	High-level group (*n* = 2198)	6.54 (2.00)	7.20	< 0.001
	Low-level group (*n* = 1107)	7.08 (2.12)		

*SD* standard deviation

Total respondents were classified into two groups (high level vs. low level) according to the median value of each type of activity engagement

### Regression analysis

Regression models are presented in Table 3. The regression model, after adjusting for other types of activity and all covariates, showed that respondents with higher social activity engagement were more likely to report fewer insomnia symptoms (*β* = − 0.04, *p* = 0.040). Likewise, those with higher cognitive activity engagement were more likely to report fewer insomnia symptoms (*β* = − 0.06, *p* = 0.007), while engaging in physical activity was not significantly associated with total insomnia symptom score (*β* = − 0.04, *p* = 0.062). Older respondents (*β* = − 0.05, *p* = 0.012) and male (*β* = − 0.10, *p* <  0.001) were more likely to report fewer insomnia symptoms. Regarding precipitating factors, respondents with chronic disease (*β* = 0.05, *p* <  0.001) and ADL difficulty (*β* = 0.11, *p* <  0.001) were more likely to report more insomnia symptoms. In case of perpetuating factors, greater loneliness (*β* = 0.20, *p* <  0.001) was associated with more insomnia symptoms.

**Table 3:** Results of multiple linear regression analysis (*N* = 3321)

	Variables	Model 1		Model 2^a^		Model 3^b^		Model 4^c^	
		***β*** (SE)	***p***	***β*** (SE)	***p***	***β*** (SE)	***p***	***β*** (SE)	***p***
Types of activity	Social activity	−0.07 (0.02)	**< 0.001**	−0.05 (0.02)	**0.008**	−0.04 (0.02)	**0.026**	− 0.04 (0.02)	**0.040**
	Cognitive activity	−0.04 (0.02)	**0.034**	−0.07 (0.02)	**0.001**	−0.06 (0.02)	**0.003**	−0.06 (0.02)	**0.007**
	Physical activity	−0.10 (0.02)	**< 0.001**	−0.09 (0.02)	**< 0.001**	−0.05 (0.02)	**0.011**	−0.04 (0.02)	0.062
Demographics	Age (years)			−0.04 (0.02)	**0.020**	−0.07 (0.02)	**< 0.001**	−0.05 (0.02)	**0.012**
	Male (ref: female)			−0.10 (0.02)	**< 0.001**	−0.09 (0.02)	**< 0.001**	−0.10 (0.02)	**< 0.001**
	Caucasian (ref: other)			−0.02 (0.02)	0.307	−0.01 (0.02)	0.765	−0.02 (0.02)	0.305
	Married (ref: other)			−0.04 (0.02)	**0.022**	−0.03 (0.02)	0.073	−0.01 (0.02)	0.506
	College or higher (ref: high school or lower)			−0.04 (0.02)	**0.018**	−**0.04 (0.02)**	**0.048**	−0.03 (0.02)	0.073
Precipitating factors	Chronic disease (ref: no)					0.06 (0.01)	**< 0.001**	0.05 (0.01)	**< 0.001**
	ADL difficulty (ref: no)					0.13 (0.02)	**< 0.001**	0.11 (0.02)	**< 0.001**
	Cognitive function					−0.04 (0.02)	0.080	−0.01 (0.02)	0.717
	Sleep disorder (ref: no)					0.03 (0.02)	0.122	0.02 (0.02)	0.208
Perpetuating factors	Loneliness							0.20 (0.02)	**< 0.001**
	Caregiving (ref: no)							0.03 (0.02)	0.063

*ADL* activities of daily living, *β* standardized coefficient, *ref* reference, *SE* standard error

Bold indicates statistical significance

^a^Adjusted for demographics

^b^Adjusted for demographics and precipitating factors

^c^Adjusted for demographics, precipitating factors, and perpetuating factors

Regression models according to the subtypes of insomnia symptoms were also presented (Table 4). Negative standardized coefficients indicate that higher values of the explanatory variable are associated with less severe insomnia symptoms. Respondents with higher social activity engagement showed lower odds of more trouble falling asleep (OR = 0.71, 95% CI *=* 0.56–0.91). Those with higher cognitive activity engagement had lower odds of more trouble falling asleep (OR = 0.77, 95% CI *=* 0.62–0.96) and more trouble with waking up too early (OR = 0.68, 95% CI *=* 0.55–0.85). Furthermore, those with higher physical activity engagement were less likely to report more nonrestorative sleep (OR = 0.69, 95% CI *=* 0.57–0.82).

**Table 4:** Results of ordinal logistic regression analyses by types of insomnia symptoms (*N* = 3321)

	Variables	Trouble falling asleep			Trouble with waking up during the night			Trouble with waking up too early			Nonrestorative sleep		
		***β*** (SE)	OR	95% CI	***β*** (SE)	OR	95% CI	***β*** (SE)	OR	95% CI	***β*** (SE)	OR	95% CI
Types of activity	Social activity	−0.06^*^ (0.02)	0.71	0.56–0.91	− 0.03 (0.02)	0.83	0.66–1.05	−0.02 (0.02)	0.88	0.69–1.12	0.01 (0.02)	1.07	0.83–1.39
	Cognitive activity	−0.05^*^ (0.02)	0.77	0.62–0.96	−0.02 (0.02)	0.92	0.74–1.14	−0.08^**^ (0.02)	0.68	0.55–0.85	−0.04 (0.02)	0.84	0.67–1.05
	Physical activity	−0.03 (0.02)	0.87	0.73–1.03	−0.00 (0.02)	0.99	0.84–1.17	0.02 (0.02)	1.07	0.90–1.28	−0.09^**^ (0.02)	0.69	0.57–0.82
Demographics	Age (years)	−0.04 (0.02)	0.99	0.98–1.00	−0.02 (0.02)	1.00	0.99–1.01	−0.02 (0.02)	0.99	0.98–1.01	−0.05^*^ (0.02)	0.99	0.98–1.00
	Male (ref: female)	−0.20^**^ (0.02)	0.47	0.40–0.55	−0.04^*^ (0.02)	0.86	0.74–0.99	−0.07^**^ (0.02)	0.78	0.66–0.91	−0.01 (0.02)	0.96	0.82–1.13
	Caucasian (ref: other)	−0.04^*^ (0.02)	0.81	0.68–0.98	0.01 (0.02)	1.04	0.87–1.24	−0.02 (0.02)	0.89	0.74–1.07	0.00 (0.02)	1.01	0.84–1.22
	Married (ref: other)	−0.01 (0.02)	0.98	0.84–1.14	−0.01 (0.02)	0.95	0.83–1.10	−0.01 (0.02)	0.97	0.83–1.12	−0.01 (0.02)	0.97	0.83–1.13
	College or higher (ref: high school or lower)	−0.04 (0.02)	0.87	0.75–1.02	−0.01 (0.02)	0.98	0.85–1.14	−0.05^*^ (0.02)	0.84	0.72–0.97	−0.03 (0.02)	0.91	0.78–1.06
Precipitating factors	Chronic disease (ref: no)	0.06^*^ (0.02)	1.73	1.19–2.50	0.04^*^ (0.02)	1.41	1.02–1.95	0.06^*^ (0.02)	1.66	1.14–2.41	0.03 (0.02)	1.27	0.87–1.87
	ADL difficulty (ref: no)	0.05^*^ (0.02)	1.31	1.08–1.57	0.07^**^ (0.02)	1.42	1.18–1.70	0.09^**^ (0.02)	1.55	1.28–1.87	0.09^**^ (0.02)	1.60	1.32–1.92
	Cognitive function	−0.02 (0.02)	0.99	0.98–1.01	0.04^*^ (0.02)	1.02	1.00–1.03	−0.01 (0.02)	1.00	0.98–1.01	−0.03 (0.02)	0.99	0.97–1.01
	Sleep disorder (ref: no)	0.04 (0.02)	1.23	1.00–1.51	0.02 (0.02)	1.13	0.92–1.38	−0.02 (0.02)	0.89	0.72–1.10	0.03 (0.02)	1.20	0.97–1.48
Perpetuating factors	Loneliness	0.14^**^ (0.02)	1.85	1.56–2.19	0.11^**^ (0.02)	1.59	1.35–1.87	0.12^**^ (0.02)	1.69	1.42–2.00	0.23^**^ (0.02)	2.79	2.35–3.32
	Caregiving (ref: no)	0.05^*^ (0.02)	1.24	1.06–1.45	0.01 (0.02)	1.05	0.90–1.22	0.01 (0.02)	1.06	0.90–1.24	0.03 (0.02)	1.13	0.96–1.33

^*^p < 0.05, ^**^p < 0.001

*ADL* activities of daily living, *β* standardized coefficient, *CI* confidence interval, *OR* odds ratio*, ref* reference, *SE* standard error

The higher value of dependent variables indicates more severe insomnia symptom

## Discussion

The purpose of this study was to examine the relationships among the different types of activity engagement and insomnia symptoms in older adults. The results of this study suggested that older adults reporting higher social, cognitive, and physical activity engagements had fewer insomnia symptoms compared to people with lower activity engagement. Furthermore, higher social, cognitive, and physical activity engagements were found to be associated with fewer insomnia symptoms, adjusting for other types of activity engagement, demographic factors, and precipitating factors. However, after including perpetuating factors (e.g., loneliness and caregiving) as covariates in the analysis, higher social and cognitive activity engagements were found to be associated with fewer insomnia symptoms, but physical activity engagement was not associated with the total insomnia symptom score.

Consistent with previous literature, the findings of this study suggest that higher social activity engagement was associated with fewer insomnia symptoms. Previous research demonstrated that older adults who reported engaging in social activities such as religious services, clubs, classes, or other organized activities were significantly less likely to show insomnia symptoms [31]. Social activity engagement may contribute to improved sleep by providing older adults with social support and encouragement to engage in positive health behaviors such as doctor visits [18] because older adults with medical problems are more likely to have sleep disturbances [32]. Moreover, previous studies have demonstrated that social activity engagement is associated with lower feelings of loneliness [11, 33], which can predict sleep complaints among older adults [10]. Therefore, social activity engagement should be promoted for better sleep.

Likewise, cognitive activity engagement should be given more attention to reduce insomnia symptoms. In this study, higher cognitive activity engagement among older adults showed a significant association with fewer insomnia symptoms. This is consistent with a previous research that showed mental activity, such as playing chess or card games that exercises memory, is associated with fewer sleep disturbances [19]. Exposure to cognitive stimuli could trigger homeostatic increases in the need for sleep [19, 34]. Moreover, cognitive activity engagement is associated with a reduced risk of incidences of dementia and cognitive impairment [35, 36], which can affect sleep complaints in older adults [37]. Especially, cognitive stimulation through cognitively challenging activities may enhance connections between neurons in the brain, which can result in improving or maintaining cognitive ability [38]. Subsequent studies need to be conducted to identify the mediating role of cognitive function in the relationship between cognitive activity and insomnia symptoms. Such studies can contribute to identifying the mechanisms that influence insomnia symptoms.

In this study, older adults with higher physical activity engagement reported fewer insomnia symptoms. However, no significant association was found between physical activity engagement and total insomnia symptom score after controlling for other types of activity and all covariates. These findings imply that physical activity engagement is significantly associated with fewer insomnia symptoms, but the extent to which physical activity engagement predicts insomnia symptoms is less than that of the other factors studied here. In particular, given that physical activity did not show a significant association with insomnia symptoms because the perpetuating factors (e.g., loneliness and caregiving) were controlled, physical activity may affect sleep by interacting with these factors rather than affecting it independently. Meanwhile, the data of this study indicate that lower physical activity engagement is associated with nonrestorative sleep among subtypes of insomnia symptoms. This is consistent with a previous research that showed positive associations between regular exercise and lower prevalence of nonrestorative sleep [39]. Considering that the positive effects of physical activity on sleep have been presented in multiple previous studies [20, 21], a further thorough investigation is needed to clarify the relationship between physical activity and insomnia symptoms.

The association between the type of activity engagement and insomnia symptoms was observed to be attenuated in this study as the covariates were controlled in the regression analyses. Considering that insomnia is a multifactorial geriatric syndrome affected by various factors [10], the association between insomnia symptoms and covariates may attenuate the effects. Sex, race, marital status, education level, chronic disease, ADL difficulty, sleep disorder, and loneliness were found to be significantly associated with insomnia symptoms in this study. Further longitudinal studies should be conducted to examine the direction of the association among these related variables, activity engagement, and insomnia symptoms.

The results of the present study have important practical implications for developing activity programs to reduce insomnia symptoms among community-dwelling older adults. Multicomponent activity programs including those activities need to be developed for older adults because social and cognitive activities are associated with fewer insomnia symptoms. In addition, it is important to support older adults to engage in activities to the extent that they are able considering that each older adult has a different level of functional impairment and specific psychosocial challenges. Likewise, older adults with greater engagement in personally meaningful activities showed better psychosocial well-being and health-related quality of life [40]. Therefore, an individualized activity program focused on individual needs and preferences should be developed.

This study has some limitations. First, variables that had been previously investigated were used because this is a secondary analysis study. For example, other sleep dimensions (e.g., sleep duration and sleep efficiency), quality of activity engagement, and specific information about the activity items were not investigated. Second, causal relationships among types of activity engagement and insomnia symptoms cannot be examined because this is a cross-sectional study. Therefore, future research should consider a prospective longitudinal design with comprehensive measures. Further, the difference in the insomnia symptoms between high- and low-level activity engagement groups showed less than 1 in all three types of activities. However, it is difficult to examine the clinical significance of activity engagement because the questionnaire for assessing insomnia symptoms used in this study was not a clinical screening tool for insomnia.

## Conclusions

The findings of this study show that older adults with higher social, cognitive, and physical activity engagements reported fewer insomnia symptoms. However, only social and cognitive activity engagements were associated with fewer insomnia symptoms after controlling for other types of activity engagement and all covariates. These results may provide practical information for developing multicomponent activity engagement strategies for improving insomnia among older adults. Particularly, these findings are noteworthy because the effect of multiple types of activity engagement on insomnia symptoms, which had yet to be studied in depth until now, was studied using a nationally representative sample of older adults.

## Supplementary Information

**Additional file 1.**

## Data Availability

The datasets from the Health and Retirement Study are publicly available online: https://hrs.isr.umich.edu/data-products

## Abbreviations

*ADL*: Activities of daily living
*CFA*: Confirmative factor analysis
*EFA*: Exploratory factor analysis
*HRS*: Health and Retirement Study
*MLR*: Maximum likelihood estimation with robust standard error
*ref*: Reference
*RMSEA*: Root mean squared error of approximation
*SRMR*: Standardized root mean square residual
*TICS*: Telephone Instrument for Cognitive Status
*WLSMV*: Weighted least square mean and variance
