# Supplementary material for: Relationships among types of activity engagement and insomnia symptoms among older adults
Source: BMC Geriatr. 2021 Jan 30;21:87. doi: 10.1186/s12877-021-02042-y (PMC7847011; doi:10.1186/s12877-021-02042-y)
Supplement: Supplementary file 1 — Additional file 1. [file 12877_2021_2042_MOESM1_ESM.pdf]

Additional file 1. Factor loadings and internal consistency reliability of the activity engagement items

(*N* = 3,321)

Items	Mean (SD)	Factor loadings		
		**Factor 1, social activity**	**Factor 2, cognitive activity**	**Factor 3, physical activity**
A. Care for a sick adult	1.38 (0.62)	0.288	−0.005	0.130
B. Do activities with grandchildren	1.76 (0.53)	0.344	0.008	0.149
C. Do volunteer work with children	1.30 (0.47)	**0.826**	−0.159	0.094
D. Do charity work	1.47 (0.52)	**0.854**	−0.059	−0.012
E. Attend an educational course	1.34 (0.48)	**0.792**	0.094	−0.043
F. Go to a sport or social club	1.54 (0.51)	**0.559**	0.154	0.088
G. Attend meetings of nonreligious organizations	1.40 (0.50)	**0.754**	0.130	−0.055
H. Pray privately	2.25 (0.76)	0.121	0.065	0.054
I. Reading	2.54 (0.59)	0.066	0.373	0.079
J. Watch television	2.87 (0.36)	−0.208	0.299	0.024
K. Do word games	1.89 (0.73)	−0.008	**0.643**	−0.073
L. Play cards or chess	1.63 (0.60)	0.132	**0.492**	−0.012
M. Writing	1.59 (0.59)	0.293	**0.403**	0.032
N. Use a computer or email	2.08 (0.88)	0.217	0.331	0.106
O. Maintenance or gardening	2.01 (0.61)	0.017	0.137	**0.426**
P. Baking or cooking	1.92 (0.53)	−0.059	0.339	0.314
Q. Knitting	1.29 (0.50)	−0.024	**0.539**	0.118
R. Work on a hobby or project	1.72 (0.58)	0.142	**0.451**	0.232
S. Play sports or exercise	1.81 (0.63)	0.130	0.055	**0.609**
T. Walk for 20 min	2.01 (0.60)	−0.043	−0.040	**0.731**
U. Participate in a community arts group	1.25 (0.44)	**0.596**	0.117	0.033
Coefficient omega of subdomain		0.89	0.72	0.68

SD standard deviation

Bold values indicate that the value of factor loading was greater than the cutoff value of 0.40.
